# Tocilizumab in treatment-naïve patients with Takayasu arteritis: TOCITAKA French prospective multicenter open-labeled trial

**DOI:** 10.1186/s13075-020-02311-y

**Published:** 2020-09-17

**Authors:** Arsene Mekinian, David Saadoun, Eric Vicaut, Sara Thietart, Bertrand Lioger, Patrick Jego, Alexandre Bleibtreu, Nicolas Limal, Jerome Connault, Jacques-Eric Gottenberg, Pauline Lhorte, Jean Pierre Bertola, Juliette Delforge, Nicole Ferreira-Maldent, Antoinette Perlat, Zohra Talib, Matthieu Vautier, Léa Savey, Isabelle Quiere, Patrice Cacoub, Olivier Fain, for the French Takayasu network

**Affiliations:** 1 Sorbonne Universités AP-HP, Hôpital Saint Antoine, service de médecine interne et Inflammation-Immunopathology-Biotherapy Department (DMU 3iD), Faculté de Médecine Sorbonne Université, F-75012 Paris, France; 2 Sorbonne Universités AP-HP, Groupe Hospitalier Pitié-Salpêtrière, Département de Médecine Interne et Immunologie Clinique, National center for Autoimmune Systemic rare disease ; National center for Autoinflammatory diseases and amyloidosis, Inflammation-Immunopathology-Biotherapy Department (DMU 3iD), INSERM, UMR_S 959, F-75013 Paris, France; 3 CNRS, FRE3632, F-75005 Paris, France; 4 CNRS, F-75013 Paris, France; 5 Unité de Recherche Clinique Saint-Louis–Lariboisière, APHP, Hôpital Saint Louis, Paris, France; 6 Service de Médecine Interne, CHU Tours, Tours, France; 7 Service de Médecine Interne, CHU Rennes, Rennes, France; 8 AP-HP, service de médecine interne, Hôpital Jean Verdier, Faculté de Paris 13, 93000 Paris, France; 9 AP-HP, service de médecine interne, Hôpital Mondor, Université Paris Est-Créteil (UPEC), Paris, France; 10 Service de Médecine Interne, CHU Nantes, Nantes, France; 11 Inserm UMR_1109, Fédération de Médecine Translationnelle, Université de Strasbourg, Strasbourg, France; 12 Service de rhumatologie, Hôpitaux Universitaires de Strasbourg, Strasbourg, France; 13 Service de Médecine Interne et vasculaire, CHU Montpellier, Montpellier, France; 14 Medical Department, Chugai Pharma France, Paris La Défense, France

**Keywords:** Takayasu arteritis, Tocilizumab, Vasculitis treatment

## Abstract

**Objectives:**

To assess long-term efficacy of tocilizumab in treatment-naive patients with Takayasu arteritis (TAK).

**Methods:**

Prospective open-labeled trial in naïve patients with TAK who received steroids at the dose of 0.7 mg/kg/day and 7 infusions of 8 mg/kg/month of tocilizumab. The primary endpoint was the number of patients who discontinued steroids after 7 infusions of tocilizumab. Secondary endpoints included disease activity and the number of relapses during 18-month follow-up.

**Results:**

Thirteen patients with TAK were included, with a median age of 32 years [19–45] and 12 (92%) females. Six (54%) patients met the primary end-point. A significant decrease of disease activity was observed after 6 months of tocilizumab therapy: decrease of median NIH scale (3 [3, 4] at baseline, versus 1 [0–2] after 6 months; *p* < 0.001), ITAS-2010 score (5 [2–7] versus 3 [0–8]; *p* = 0.002), and ITAS-A score (7 [4–10] versus 4 [1–15]; *p* = 0.0001)]. During the 12-month follow-up after tocilizumab discontinuation, a relapse occurred among 5 patients (45%) out of 11 in which achieved remission after 6 months of tocilizumab.

**Conclusion:**

Tocilizumab seems an effective steroid sparing therapy in TAK, but maintenance therapy is necessary.

**Trial registration:**

ClinicalTrials.gov NCT02101333. Registered on 02 April 2014.

## Key messages

What is already known about this subject?

- Tocilizumab is an effective sparing therapy for refractory and steroid-dependent Takayasu arteritis

What does this study add?

- This is the first trial of biologics, in particular tocilizumab in treatment-naïve Takayasu patients, with steroid discontinuation strategy after 6 months of combined therapy

How might this impact on clinical practice?

- Tocilizumab addition to steroids in treatment-naïve Takayasu patient’s is highly effective, but maintenance therapy is necessary.

## Introduction

Takayasu arteritis (TAK) is a chronic primary vasculitis that affects large vessels, particularly the aorta and its main branches [1]. Its main complications are consecutive to vascular inflammation, which could lead to arterial stenosis, aneurisms, and thrombosis. The best therapeutic strategy in TAK remains to be determined. Indeed, steroids are the cornerstone of treatment for TAK and allow 71% of sustained remission (defined by a use of less than 10 mg/day of prednisone) [2]. Despite this high sustained remission rates, the relapse rates are at 46% at 5 years [2]. Data on the benefit of biological-targeted therapies and disease-modifying anti-rheumatic drugs (DMARDs) is growing, with small case-series describing the use of steroid-sparing agents such as azathioprine, methotrexate, or mycophenolate mofetil [3–5]. We recently reported a French multicenter study showing the benefit of biologic-targeted treatments in refractory TAK, with higher relapse-free and vascular event-free survivals comparatively with DMARDs [6].

Increasing evidence suggests that interleukin-6 plays a role in the pathogenesis of TAK. Presence of vascular inflammatory infiltrates rich in T cells producing interleukin-6 was found in aortic wall samples of patients with TAK [7].

Rapid and sustained remission under tocilizumab therapy has been described, notably among patients with refractory TAK [8–13]. These findings should be interpreted with caution, as most studies were case reports or small series, and vascular progression under tocilizumab treatment has also been described [8–13]. A recent randomized trial failed to demonstrate the benefit of tocilizumab, compared to placebo, on relapse-free survival among patients with refractory TAK [14]. Efficacy of biological-targeted therapies, such as TNFα antagonists and tocilizumab, was mainly reported in patients with refractory TAK. The effect of tocilizumab on patients with treatment-naïve TAK remains unclear.

In this multicenter, prospective, open-labeled trial, we aim to evaluate the benefit of adding tocilizumab to steroids in treatment-naïve patients with TAK, on discontinuation of steroids after 6 months of tocilizumab treatment, and to assess relapse-free survival following tocilizumab discontinuation.

## Patients and methods

Patients were enrolled if they were aged 18 years or older, with a diagnosis of TAK (according to the ACR criteria and/or Ishikawa criteria modified by Sharma) established between October 2014 and July 2017. All patients were treatment-naive or free from any immunosuppressive therapy (DMARDs and biological-targeted therapies) for at least 4 months. If previously initiated, steroids must have been started within the month preceding inclusion.

The trial is registered on ClinicalTrials (NCT02101333) and could be find at https://clinicaltrials.gov/ct2/show/NCT02101333?cond=takayasu&draw=2&rank=8; registration date 02/04/2014, registration number *NCT02101333*). All patients gave written informed consent and consent for publication. The trial conformed the ethical guidelines of the Declaration of Helsinki and was approved by the Institutional Review Boards of the Ethic committee of “Cochin Hospital University” (CPP Ile de France, Cochin Hospital, 02/02/2014)(N° ID-RCB 2017-AO3380-53).

### Study design

This prospective open-labeled trial was designed to evaluate the effect of tocilizumab to discontinue steroids after 6 months of tocilizumab therapy, among treatment-naïve patients with TAK. Induction therapy consisted of corticosteroids at a dose of 0.7 mg/kg/day and 7 infusions of tocilizumab at a dose of 8 mg/kg/month. Steroids were decreased using a predefined regimen schedule over the first 6 months of tocilizumab therapy (Supplementary Table 1).

### Assessment and definitions

The primary endpoint was the number of patients that achieved steroid discontinuation after 7 infusions of tocilizumab.

Secondary endpoints included disease activity; clinical, biological, and radiological responses at 3, 6, 9, 12, 15, and 18 months; rates of sustained remission; number of relapses; time with sustained remission; cumulative steroid doses; frequency of ischemic vascular complications and interventions; and safety.

Clinical response was defined as the absence of new symptoms and/or disappearance of all previous symptoms. Biological response was defined as normalization of all acute-phase reactants, including erythrocyte sediment rate (ESR), C-reactive protein (CRP) and fibrinogen, or a minimum 50% decrease of at least 2 of these markers. Radiological response was defined as the absence of arterial progression at repeated imaging at 6 months after tocilizumab initiation as compared to baseline. Disease activity was defined using NIH and ITAS-2010 scales. Briefly, disease was considered as active if NIH score was of 2 or more, and inactive otherwise; sustained remission was defined as a NIH < 2 with a prednisone dosage < 10 mg/day. Disease activity was also subjectively evaluated as active, stable, or remission, using practitioner’s judgment, independently from NIH scale and other objective laboratory markers. Relapse was defined as the occurrence, among patients that achieved remission, of disease activity requiring a change in treatment regimen. Ischemic vascular events and/or the need for a vascular intervention were recorded during the 18-month follow-up.

Safety was assessed as the incidence and severity of adverse events (AEs), drug reactions, and alterations of laboratory findings.

### Statistical analysis

Response rates were of 70% using tocilizumab in patients with refractory TAK. We hypothesized that remission would be achieved among 50% of patients after 6 months of tocilizumab therapy, with an estimated precision of ± 25%.

Data are presented as medians with ranges for continuous variables and frequencies with percentages for qualitative variables. Fisher’s exact test was used to compare qualitative variables and the Wilcoxon rank test to compare continuous variables. All tests were two-sided, and a *p* value < 0.05 was considered as statistically significant. Statistical analyses were performed using R software (version 3.1.0).

## Results

### Patient characteristics

Thirteen patients with TAK were included, with a median age of 32 years [19–45], and 12 (92%) females. Patients’ geographical origins included 7 (54%) Caucasians, 4 (31%) North-Africans, and 2 (15%) of other origins. Baseline cardiovascular risk factors were arterial hypertension in 2 patients (15%), hyperlipidemia in 2 patients (15%), and tobacco use in 3 patients (23%), with no type 2 diabetes nor cardiovascular heredity. Median body mass index was 25 kg/m^2^ [20–32]. Two patients had an associated autoimmune disease, which were systemic lupus erythematosus and a Graves’ disease. All patients were treatment-naïve, except for 4 patients who previously received DMARD therapy (2 patients under azathioprine and 2 under methotrexate), but were free of DMARDs for at least 24 months and never received any other therapies, in particular biologics. The 4 patients who previously received DMARD therapy were DMARD-free since more than 2 years and have relapsing active TAK disease. Median time between diagnosis and inclusion was 8 months [0.7–185].

### Efficacy of tocilizumab at 6 months

**Fig. 1:**
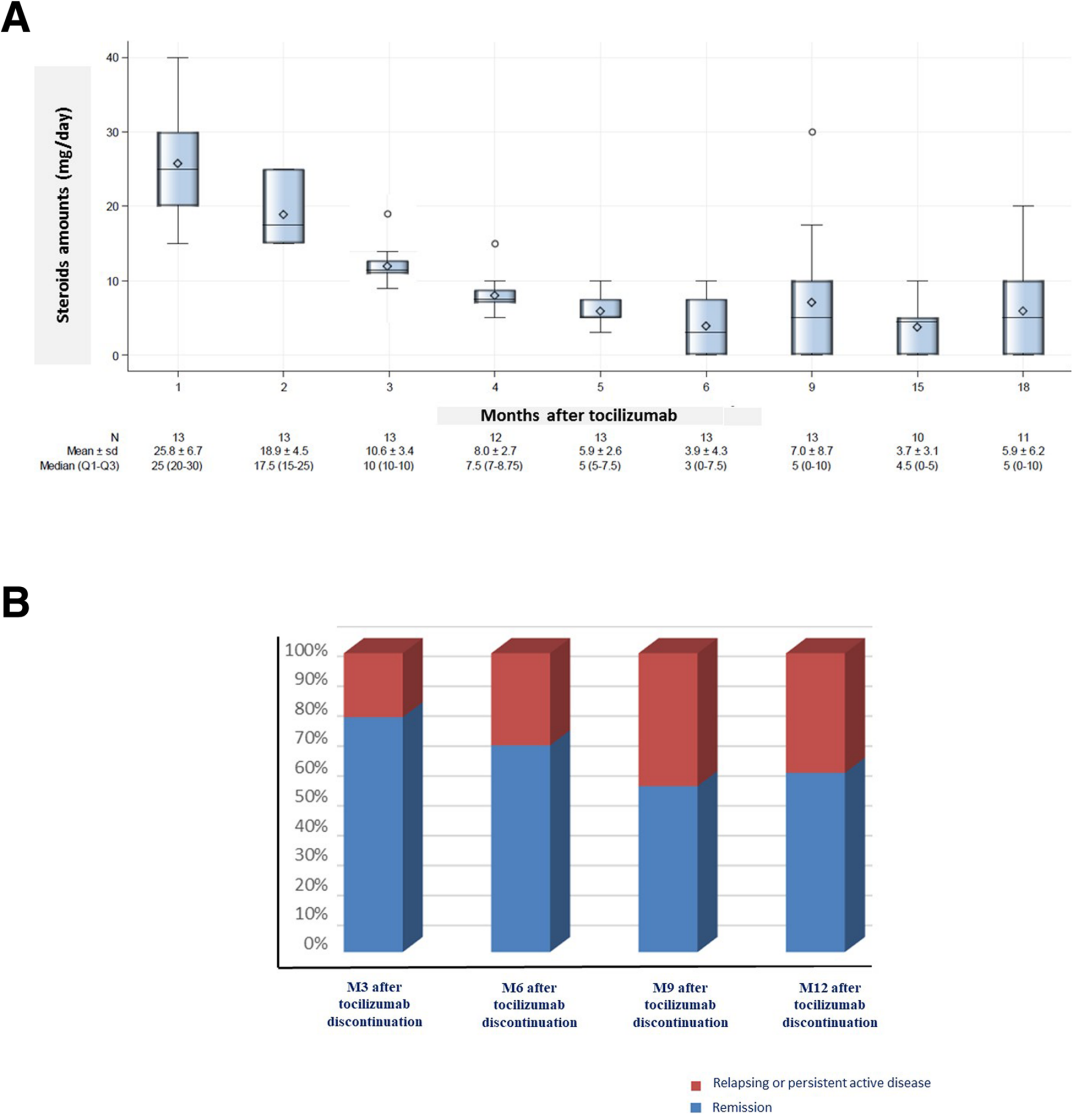
TOCITAKA trial data of steroids sparing effect and relapse rates after 6-month tocilizumab therapy. **a** Steroids amounts during the 18-month follow-up in TOCITAKA trial. **b** Frequencies of relapse after 6-month tocilizumab induction therapy, represented as cumulative proportion of patients in remission and with relapsing-persistent TAK disease at months 3, 6, 9, and 12 after the tocilizumab discontinuation

The primary endpoint (i.e., TAK remission and steroid withdrawal after 7 tocilizumab infusions) was reached in 6/13 (54%) of patients. Overall, TAK remission after 7 tocilizumab infusions was obtained in 11 (85%) patients at 6 months. Among the 5 remaining patients who continued steroids, 3 had a prednisone-equivalent dosage < 5 mg/day. Eleven patients (85%) achieved sustained remission rates after 6 months, with a NIH < 2 and prednisone doses < 10 mg/day, and among them 9 patients (69%) had prednisone doses < 7.5 mg/day. Patients undergoing steroid therapy significantly decreased between baseline (13 patients, 100%) and 6 months of tocilizumab therapy (6 patients, 54%, *p* = 0.015), as shown in Fig. 1a. NIH scales also significantly decreased between baseline and 6 months of tocilizumab therapy (3 [3–4] at baseline versus 1 [0–2] at 6 months; *p* < 0.001), as well as ITAS-2010 (5 [2–7] versus 3 [0–8]; *p* = 0.002), and ITAS-A score (7 [4–10] versus 4 [1–15]; *p* = 0.0001). Baseline practitioners’ subjective scale evaluated that 13 patients (100%) had active TAK, versus 2 patients (15%) after 6 months (*p* < 0.001) (Table 1). Acute-phase reactants significantly decreased after 6 months of tocilizumab therapy (Table 1).

**Table 1:** Patients’ characteristics at initiation of tocilizumab and during follow-up

	At initiation of tocilizumab ***N*** = 13	At 1 month ***N*** = 13	At 2 months ***N*** = 13	At 3 months ***N*** = 13	At 4 months ***N*** = 12	At 5 months ***N*** = 13	At 6 months ***N*** = 13	At 9 months ***N*** = 13	At 12 months ***N*** = 12	At 15 months ***N*** = 10	At 18 months ***N*** = 11
**Vascular manifestations**	13 (100)	9 (69)	10 (100)	8 (62)	4 (33)	7 (54)	7 (54)	7 (54)	7 (58)	4 (40)	4 (36)
**-**Arm claudication	5 (38)	3 (23)	4 (30)	2 (15)	2 (15)	4 (30)	3 (23)	4 (30)	2 (17)	2 (20)	2 (18)
**-**Lower limb claudication	3 (23)	1 (8)	0	1 (8)	0	0	1 (8)	2 (15)	0	0	0
**-**Anisotension	7 (54)	7 (54)	8 (62)	4 (30)	3 (23)	5 (38)	4 (30)	4 (30)	5 (42)	3 (30)	3 (27)
**-**Carotidynia	9 (69)	1 (8)	3 (23)	1 (8)	0	2 (15)	3 (23)	3 (23)	3 (23)	2 (20)	1 (9)
**Constitutional manifestations**	5 (38)	2 (15)	3 (23)	1 (8)	2 (17)	2 (15)	4 (33)	1 (8)	1 (8)	2 (20)	0
**-**Arthromyalgias	3 (23)	0	1 (8)	1 (8)	0	1 (8)	2 (15)	4 (31)	0	0	0
**-**Fever/asthenia	3 (23)	2 (15)	1 (8)	1 (8)	2 (15)	2 (15)	4 (31)	1 (8)	1 (8)	1 (8)	0
**NIH score**	3 [3–4]	–	–	1 [0–2]	–	–	1 [0–2]	1 [0–3]	1 [0–4]		1 [0–3]
**ITAS 2010/ITAS-A**	5 [2–7]/7 [4–10]	–	–	2 [0–5]/2 [0–5]	–	–	3 [0–8]/4 [1–15]	3 [0–8]/4 [0–11]	2 [0–4]/2.5 [0–34]		1 [0–7]/3 [0–9]
**Subjective disease activity by practitioner**											
**-Remission**	–	5 (38)	5 (38)	7 (54)	7 (54)	9 (69)	8 (62)	7 (54)	7 (58)	6 (60)	7 (64)
**-Active**	13 (100)	0	0	1 (8)	0	2 (15)	2 (15)	3 (23)	2 (16)	4 (40)	4 (36)
**-Stable**	–	7 (54)	7 (54)	5 (38)	6 (50)	2 (15)	2 (15)	3 (23)	3 (25)	0	0
**C-reactive protein (mg/L)**	16 [3–98]	1 [0–14]	1 [0–10]	0 [0–2]	1 [0–17]	1 [0–17]	1 [0–12]	4 [0–53]	3 [0–28]	10 [0–42]	14 [0–45]
**Fibrinogen (g/l)**	5 [2.8–8]	2 [1–5]	2 [2–6]	2 [0–4.5]	2 [1.5–5]	2 [1.5–5]	2 [1–3]	3 [2–7]	3 [1–5]	4 [2–7]	5 [2–6]
**Prednisone (*n*; %)**	13 (100)	13 (100)	13 (100)	13 (100)	12 (100)	13 (100)	7 (54)	7 (54)	6 (50)	7 (70)	7 (64)
**Prednisone (mg/day)**	45 [35–65]	25 [15–40]	20 [15–30]	10 [10–25]	7.5 [5–17.5]	5 [2.5–30]	5 [0–10]	3 [0–30]	2.5 [0–15]	3.5 [0–10]	3 [0–10]
**Immunosuppressive drugs**	0	0	0	0	0	0	0	MTX (*n* = 2) /Ada (*n* = 1)/Inf (*n* = 1)	MTX (*n* = 2)/Ada (*n* = 1)/Inf (n = 1)/Toci (*n* = 1)	MTX (*n* = 2)/MTX+ Ada (*n* = 1)/MTX+ Inf (*n* = 1)/Toci (*n* = 2)	MTX + Ada (*n* = 2) /Uste (*n* = 1)/MTX + Toci (*n* = 1)/Toci (*n* = 2)/Aza (*n* = 1)
**NIH < 2**											
**+prednisone < 7.5 mg/day**	**–**	–	–	–	–	–	9 (69)	7 (54)	6 (50)	9 (90%)	4 (36)
**< 10 mg/day**							11 (85)	9 (69)	9 (75)	10 (100)	6 (55)

Values are presented as medians [ranges] and numbers (frequencies)

*Inf* infliximab, *Toci* tocilizumab, *Uste* ustekinumab, *Ada* adalimumab, *Aza* azathioprine, *MTX* methotrexate

*p < 0.0001 between baseline and all visits during the follow-up (Kruskal-Wallis tests or Fisher’s test)

### Outcome after tocilizumab discontinuation from 6 to 18 months

All patients discontinued tocilizumab after 7 infusions, and no other immunosuppressive drugs was introduced, except for 1 patient which received methotrexate. After 9 and 12 months, respectively 7 (54%) and 6 (50%) patients achieved remission with less than 7.5 mg/day of prednisone, and 9 (69%) and 9 (75%) with doses < 10 mg/day (Table 1). After 12 months of tocilizumab initiation, among patients who discontinued steroid-therapy, 2 (33%) had an active disease and 3 (50%) had biological activity, whereas none of the patients who continued steroid therapy had disease or biological activity (Supplementary Table 2).

Among the 11 patients who achieved 6-month remission, a relapse occurred within 12 months of tocilizumab discontinuation among 5 patients (45%): 3 during the first 3 months and 2 within 6 months after the last tocilizumab infusion (Fig. 1b). During 18-month follow-up, 4 patients (36%) remained free from any treatment, 2 (18%) were treated with steroids alone with doses under 7.5 mg/day, all others were received tocilizumab or other therapies (Table 1).

Among the 2 patients who were non-responders after 6 months of tocilizumab therapy, 1 achieved remission after switching to infliximab and methotrexate, and the other achieved remission after switching to several other biological-targeted therapies. No significant vascular complications or interventions were noted during follow-up.

### Safety

Adverse effects were reported in 9 patients (69%) during the 6-month tocilizumab therapy: rhinopharyngitis and otitis (*n* = 3), viral gastroenteritis (*n* = 2), and asymptomatic neutropenia (> 500/mm^3^), urinary tract infection, thoracic zona, and acute pancreatitis (*n* = 1 for each). No severe AEs were considered related to study treatment, and none required tocilizumab interruption or dose reduction. No deaths have occurred during the study period.

## Discussion

To our knowledge, this is the first prospective trial aiming to evaluate the benefit of a combination of biological-targeted therapy with steroids, to allow steroid discontinuation among treatment-naïve patients with TAK. The 2 key messages are as follows: (1) tocilizumab combined to steroids in treatment-naïve patients with TAK is highly effective, with 85% remission rate and 54% of steroid discontinuation after 6 months of therapy, and (2) despite the high remission rates obtained using tocilizumab, relapse rates were of 45% after discontinuation. These findings should be considered with caution in the absence of randomized placebo-controlled group treated by steroids alone.

Previous studies on tocilizumab efficacy mainly reported data on patients with refractory TAK. The only randomized trial, which compared 36 patients who received subcutaneous tocilizumab versus placebo, showed that time to remission was not significantly different, but relapse time was longer in the tocilizumab group [14]. Other retrospective studies have reported efficacy of tocilizumab, but once again mainly in patients with refractory TAK [15–18]. A recent literature review of 105 patients with TAK treated by tocilizumab reported overall clinical and radiological response rates of 85.7% and 65.2%, respectively [16]. We recently reported data from a French nationwide study describing the use of infliximab and tocilizumab, and similar risks of vascular complications were found [6]. Overall incidence of vascular complications reached more than 50% after 5 years of follow-up of patients with TAK treated with DMARDs [19]. However, no vascular events occurred in our study during follow-up. Here, we report for the first time data on tocilizumab for treating patients with treatment-naïve TAK, and we show a good remission rate, with 54% of steroid-free patients after 6 months. Considering treatment-naïve patients with TAK, the overall response rate using tocilizumab in addition to steroids in our study seemed higher than with steroids alone from previous literature data (85% versus 40–60%, respectively) [1]. In DMARD-treated patients, a recent meta-analysis showed 58% of remission rates and 54% of relapse rates in steroid-dependent or refractory TAK [20]. Even overall response rates are better in our study with treatment-naïve TAK patients, the relapse rates seem to be similar to DMARD-treated TAK and highlight the need of maintenance therapy, and our study do not allow the comparison of biologics versus DMARDs therapies.

Relapse is a particularity challenging issue and occurred among 45% of patients with TAK, despite high initial remission rates. After remission induced by tocilizumab, 36% remained treatment-free during the 18-month follow-up. Literature has mainly reported studies on steroid-dependent or refractory patients with TAK, which makes it difficult to evaluate relapse-free survival rates of treatment-naïve patients. Maintenance regimen therapies still need to be determined and well-designed prospective studies on patients with treatment-naïve TAK are necessary.

Safety of tocilizumab is well-established in several autoimmune conditions. In our study, adverse effects occurred in 69% of patients, but none were severe, nor have induced tocilizumab discontinuation, or therapy-related deaths [16].

Our study has several limitations, such as the small number of included patients and the absence of a double-blinded placebo control group. Despite these limitations, this trial is the first to show that a high response rate can be achieved by adding tocilizumab to conventional steroids and that 36% of patients with TAK will remain without treatment during at least 18 months.

## Conclusion

This multicenter study is the first trial to assess, in treatment-naïve patients with TAK, the value of adding tocilizumab to steroids. However, the high steroid-free remission rates after 6 months of tocilizumab therapy should be balanced by high relapse rates and should consider the absence of randomized placebo-controlled group. Further studies among treatment-naïve patients with TAK are necessary to determine the best maintenance therapy strategy.

## Supplementary information

**Additional file 1 : Supplementary Table 1**. Steroids tapering schema from the baseline to 6-months after tocilizumab initiation. **Supplementary Table 2**. Clinical, biological and remission rates of patients, which withdrew or continued steroids after 6 months of tocilizumab therapy.

## Data Availability

Not applicable.

## Abbreviations

CRP: C-reactive protein
DMARDs: Disease-modifying anti-rheumatic drugs
ESR: Erythrocyte sediment rate
ITAS: International Takayasu arteritis scale
MTX: Methotrexate
NIH: National Institutes of Health
TAK: Takayasu arteritis
