# Supplementary material for: Tocilizumab in treatment-naïve patients with Takayasu arteritis: TOCITAKA French prospective multicenter open-labeled trial
Source: Arthritis Res Ther. 2020 Sep 17;22:218. doi: 10.1186/s13075-020-02311-y (PMC7500024; doi:10.1186/s13075-020-02311-y)
Supplement: Supplementary file 1 — Additional file 1 : Supplementary Table 1. Steroids tapering schema from the baseline to 6-months after tocilizumab initiation. Supplementary Table 2. Clinical, biological and remission rates of patients, which withdrew or continued steroids after 6 months of tocilizumab therapy. [file 13075_2020_2311_MOESM1_ESM.docx]

**Tocilizumab in treatment-naïve patients with Takayasu arteritis: TOCITAKA French prospective multicenter open-labelled trial.**

Arsene Mekinian^1,^ MD, PhD^,^ David Saadoun^2,^ MD, PhD, Eric Vicaut^3^, MD, PhD, Sara Thietart^1^, MD, Bertrand Lioger^4^, MD, Patrick Jego^5^,  MD, PhD, Alexandre Bleibtreu^6^, MD, PhD, Nicolas Limal^7^, MD, Jerome Connault^8^, MD, PhD, Jacques-Eric Gottenberg^9^, MD, PhD, Pauline Lhorte^10,^ MD, Jean Pierre Bertola^11^, MD, Juliette Delforge^6^, MD, Nicole Ferreira-Maldant^4^, MD, Antoinette Perlat^5^, MD, Zohra Talib^3^, Matthieu Vautier^2^, MD, Léa Savey^2^, MD, Isabelle Quiere^10^, Patrice Cacoub*^2^, MD, PhD, Olivier Fain*^1^, MD, for the French Takayasu network.

^1^Sorbonne Universités AP-HP, Hôpital Saint Antoine, service de médecine interne et Inflammation-Immunopathology-Biotherapy Department (DMU 3iD), Faculté de Médecine Sorbonne Université, F-75012, Paris, France

^2^ Sorbonne Universités AP-HP, Groupe Hospitalier Pitié-Salpêtrière, Département de Médecine Interne et Immunologie Clinique, National center for Autoimmune Systemic rare disease ; National center for Autoinflammatory diseases and amyloidosis, Inflammation-Immunopathology-Biotherapy Department (DMU 3iD), INSERM, UMR_S 959, F-75013, Paris, France, CNRS, FRE3632, F-75005, Paris, F-75013, Paris, France.

^3^ Unité de Recherche Clinique Saint-Louis–Lariboisière, APHP, Hôpital Saint Louis, Paris, France

^4^ Service de Médecine Interne, CHU Tours, Tours, France.

^5^Service de Médecine Interne, CHU Rennes, Rennes, France.

^6^AP-HP, service de médecine interne, Hôpital Jean Verdier, Faculté de Paris 13, 93000, Paris, France

^7^AP-HP, service de médecine interne, Hôpital Mondor, Université Paris Est-Créteil (UPEC), France

^8^Service de Médecine Interne, CHU Nantes, Nantes, France.

^9^Inserm UMR_1109, Fédération de Médecine Translationnelle, Université de Strasbourg ; Service de rhumatologie, Hôpitaux Universitaires de Strasbourg, Strasbourg, France

^10^Service de Médecine Interne et vasculaire, CHU Montpellier, Montpellier, France.

^11^Medical Department, Chugai Pharma France, Paris La Défense, France

*equally contributed

**Supplementary Table 1. Steroids tapering schema from the baseline to 6-months after tocilizumab initiation.**

| **Steroids decrease proposal** | **Example for 60 kg** | **70 kg** |
| --- | --- | --- |
| M0: 0.7 mg/kg | 40 mg | 50 mg |
| W2: 0.5 mg/kg | 30 mg | 40 mg |
| W4: 0.4 mg/kg | 25 mg | 35 mg |
| W6: 0.3 mg/kg | 20 mg | 30mg |
| W8: 0.25 mg/kg | 15 mg | 25 mg |
| W10: 0.2 mg/kg | 10 mg | 20 mg |
| W12: 0.15 mg/kg | 10 mg | 15 mg |
| W14: 0.125 mg/kg | 7.5 mg | 10 mg |
| W18: 0.1 mg/kg | 5 mg | 7.5 |
| W20: | 1 mg/10 days | 5 |
| W22: | 1 mg/10 days | 1 mg/10 days |
| W24: Steroids discontinuation | 0 | 0 |

**Supplementary Table 2. Clinical, biological and remission rates of patients, which withdrew or continued steroids after 6 months of tocilizumab therapy.**

|  | **All**  **N = 11** | **Steroids withdrawn**  **after 6 months**  **N = 6** | **Continuation of steroids**  **after 6 months**  **N = 5** | **p** |
| --- | --- | --- | --- | --- |
| **Disease clinical activity** |  |  |  |  |
| - At month 9 | 3 (27) | 1 (16) | 2 (40) | 0.5 |
| - At month 12 | 2 (20) | 2 (33) | 0 | 0.45 |
| - At month 15 | 1( 12) | 1 ( 20) | 0 | 1 |
| - At month 18 | 1 (11) | 1 (17) | 0 | 1 |
| **Biological activity** |  |  |  |  |
| - At month 9 | 3 (27) | 3 (50) | 0 | 0.18 |
| - At month 12 | 3 (50) | 2 (50) | 0 | 0.45 |
| - At month 15 | 3 (25) | 2 (40) | 0 | 0.45 |
| - At month 18 | 3 (33) | 3 (50) | 0 | 0.18 |
| **Sustained remission** |  |  |  |  |
| - At month 9 | 8 (72) | 5 (83) | 3 (60) | 0.54 |
| - At month 12 | 8 (72) | 3 (50) | 5 (100) | 0.5 |
| - At month 18 | 5 (56) | 2 (33) | 3 (100) | 0.6 |
